# Serum Müllerian inhibiting substance levels are lower in premenopausal women with breast precancer and cancer

**DOI:** 10.1186/1756-0500-4-152

**Published:** 2011-05-26

**Authors:** Andrew C McCoy, Beth Kliethermes, Ke Zhang, Wenyi Qin, Robert Sticca, Michael Bouton, Edward R Sauter

**Affiliations:** 1 Department of Surgery, University of North Dakota School of Medicine, Grand Forks, ND, USA; 2 Department of Pathology, University of North Dakota School of Medicine, Grand Forks, ND, USA

## Abstract

**Background:**

In preclinical studies, müllerian inhibiting substance (MIS) has a protective affect against breast cancer. Our objective was to determine whether serum MIS concentrations were associated with cancerous or precancerous lesions. Blood from 30 premenopausal women was collected and serum extracted prior to their undergoing breast biopsy to assess a suspicious lesion found on imaging or physical examination. Based on biopsy results, the serum specimens were grouped as cancer (invasive or ductal carcinoma *in situ*), precancer (atypical hyperplasia or lobular carcinoma *in situ*), or benign.

**Findings:**

Serum from women with cancer and precancer (p = .0009) had lower MIS levels than serum from women with benign disease.

**Conclusion:**

Our findings provide preliminary evidence for MIS being associated with current breast cancer risk, which should be validated in a larger population.

## Introduction

Müllerian inhibiting substance (MIS), also known as anti-müllerian hormone, is produced by the granulosa cells of growing ovarian follicles. The level of MIS varies throughout a woman's lifetime. MIS is detectable from birth, but increases substantively at puberty. After puberty, MIS slowly decreases until it becomes undetectable after menopause [1]. MIS levels have recently been linked to breast cancer risk [2]. MIS, a member of the transforming growth factor (TGF)-β superfamily, is a 140 kDa dimeric glycoprotein that binds to the MIS type II receptor in breast tissue [3,4]. MIS inhibits the growth of cultured breast cancer cells through G1 arrest and the induction of apoptosis [5,6]. An eight-fold increase in the ratio of apoptotic cells in murine mammary tissue was observed after MIS injection [6]. Based on these observations, our hypothesis was that MIS would decrease breast cancer risk in premenopausal women, and that MIS serum levels would be different in women with benign breast disease vs. those with precancer or cancer. The objective of our study was to determine if MIS predicted the benign or malignant nature of a breast lesion requiring biopsy.

### Material and methods

After Institutional Review Board approval, informed consent was obtained. Thirty blood samples were prospectively collected in serum separator tubes from premenopausal women 38-50 years of age scheduled to undergo diagnostic breast biopsy to determine the benign or malignant nature of a suspicious breast lesion identified on imaging or physical examination. Pregnant or lactating women, as well as women receiving chemotherapy or radiation therapy, were not eligible. The cohort of randomly collected samples included 14 from women diagnosed with cancer, 8 with precancer and 8 with benign lesions collected between November 2001 and May 2008. Participant samples were classified as "cancer" if the diagnostic biopsy demonstrated ductal carcinoma *in situ *or invasive breast cancer; "precancer" if the biopsy demonstrated atypical hyperplasia or lobular carcinoma *in situ*; or "benign" if the biopsy was benign. The blood was spun down, serum decanted and snap frozen at -80°C until analysis.

Samples were analyzed as a single batch by a scientist (WQ) blinded as to sample diagnosis using an MIS ELISA kit (catalog no. DSL-10-14400) from Diagnostic Systems Laboratories (Webster, TX), according to the manufacturer's instructions. The scientist analyzing the samples was blinded regarding the participant's diagnosis. The kit has a detection limit of 0.006 ng/mL. An intra-assay CV was calculated for all samples with all values that were at least three times the detection limit of the assay. The mean CV was 4.0% (range 0-16.1%). Values below the detection limit were assigned a value of half the detection limit, 0.003 ng/ml. The raw data were right-skewed and not normally distributed based on the Shapiro-Wilk test. Therefore, the data were logarithm transformed, checked using the same test, and found not to be significant. The transformed data were then analyzed. The distribution of MIS levels in the three risk groups (benign, precancer, and cancer) were assessed using analysis of variance (ANOVA). Pairwise differences between risk groups were analyzed using Fisher's LSD (least significant difference). Time from sample collection to analysis, participant age, age at menarche, body mass index (BMI), whether the participant had a first degree relative with breast cancer, current or ever use of oral contraceptive pills (OCPs) were assessed for their potential cofounding effects. All analyses were conducted using SAS JMP software.

## Results

**Figure 1:**
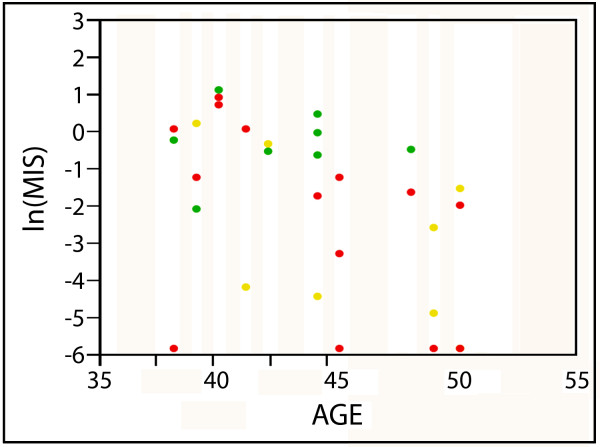
**Natural log (ln) levels of MIS in serum collected from premenopausal women prior to diagnostic breast biopsy**. Colors designate individuals with the following diagnoses: green-benign; yellow-precancer; red-cancer. Values below the detection limit were assigned a value of half the detection limit.

Participant demographics are summarized in Table 1. Twenty-nine of the 30 women reported having a period within a year prior to enrollment. The one woman who did not had undergone uterine ablation and was 45 years old at the time of enrollment, with intact ovaries. Due to her age, she was classified as premenopausal. Time from sample collection to analysis, participant age, age at menarche, those with a first degree relative with breast cancer, ever or current OCP use and BMI did not significantly differ based on pathologic diagnosis. MIS was inversely associated with risk of breast cancer (p = .04) or precancer (p = .02) compared to women who had a benign biopsy (Table 1 Figure 1). Moreover, MIS was highly inversely associated with the presence of any disease (cancer or precancer) vs. benign (p = .0009). We evaluated if there were outliers that made the comparison between disease vs. benign not significant. No such outliers were identified.

**Table 1:** Demographics^1^

	Benign	Precancer	Cancer
***Mean ± SD***			

**Sample size**	8	8	**14**

**Time from sample collection to analysis (years)**	3.02 *± *1.04	4.19 *± *2.40	**2.24 *± *0.49**

**Participant age**	42.4 *± *3.3	45.5 *± *4.5	**43.7 *± *4.4**

**Age at menarche**	13.1 ± 1.2	12.6 ± 1.0	**12.6 ± 1.6**

**BMI **	30.7 ± 6.9	30.7 ± 10.0	**27.4 ± 9.1**

**MIS (ng/mL)**	1.07 ± 0.90	0.30 ± 0.50	**0.58 ± 0.80**

**ln(MIS (ng/mL)**	-0.27 ± 0.93	-2.91 ± 2.24	**-2.31 ± 2.55**

***Frequency (%)***			

**1st degree relative with breast cancer **	1 (12.5%)	3 (37.5%)	**2 (14.3%)**

**Current OCP use **	1 (12.5%)	0 (0%)	**2 (14.3%)**

**History of OCP use**	**4 (50%)**	**6 (75%)**	**12 (85.7%)**

1: Results for time to sample collection, participant age, age at menarche, body mass index (BMI), and mullerian inhibiting substance (MIS) are mean +/- standard deviation. Results for 1^st ^degree relative with breast cancer, current and history of oral contraceptive pill (OCP) use are frequency (%).

## Discussion

Since the primary purpose of MIS in males is the regression of Mullerian structures through the induction of apoptosis [7], it follows that apoptosis induced by cells sensitive to MIS in females may protect against malignant transformation. Breast ductal epithelial cells have MIS receptors which upon stimulation induce apoptosis and inhibit growth [5,6]. We observed a negative association of MIS concentration with cancerous and precancerous breast lesions in premenopausal women 38-50 years of age. This age range was chosen because breast precancer and cancer is uncommon in women less than 35 years of age [8], and is consistent with the upper age bound used in a prior report on MIS ^2^.

Our observation of lower MIS levels in women with breast precancer and cancer is consistent with both *in vitro *and animal studies, which suggest that MIS has a cancer preventive effect [3,5,6], but is in contrast to an epidemiologic study [2] which suggested that increased MIS levels in healthy women were associated with an increased risk of breast cancer in the future. In that report, the journal editors noted that the association of increased serum MIS concentrations with increased future risk of developing breast cancer is in contrast to previous preclinical findings and what is known about the mechanisms of MIS on breast physiology, which would predict that high MIS concentrations would be associated with lower risk. The author of the epidemiologic study noted that their cohort of case participants had a lower frequency of oral contraceptive use compared to controls, which is at odds with most reports [9]. On the other hand, the OCP use in our precancer and cancer participants was not lower than controls and trended higher in the cancer group, which may in part explain the difference in our results. Additionally, our observations regarding MIS are for current risk, compared to the future risk that was evaluated in the epidemiologic study.

There are limitations to the current study. The first is the limited sample size. A second is that MIS, though associated with disease, was not 100% accurate in differentiating women with precancer or cancer from those without. As such, it is likely that MIS, if validated as associated with current disease in the breast, will need to be combined with other biomarkers for optimal disease prediction. We evaluated whether there was an outlier which might have influenced the association of MIS with disease, but could find none. Our findings provide preliminary evidence for MIS being associated with current breast cancer risk, which should be validated in a larger population.

## List of Abbreviations

ANOVA: analysis of variance, BMI: body mass index; ELISA: enzyme linked immunosorbant assay; LSD: least significant difference; MIS: müllerian inhibiting substance; OCPs: oral contraceptive pills

## Competing interests

The authors declare that they have no competing interests.

## Authors' contributions

ACM helped with study design and manuscript preparation, BK gathered the data and prepared it for statistical review, WQ performed the MIS analysis. RS and MB helped with manuscript preparation, ERS designed the study, collected the samples and supervised specimen analysis and manuscript preparation. All authors read and approved the final manuscript.
